# Medical College Notes

**Published:** 1897-10

**Authors:** 


### Medical College Notes.

Buffalo University Medical College opened its regular session on Monday, September 13, 1897. Dr. Montgomery A. Crockett delivered the introductory lecture and the assembled students gave an enthusiastic reception to Dr. Roswell Park upon that his first appearance amongst them since his recovery.

Niagara University Medical College inaugurated its scholastic year on Tuesday, September 28, 1897. Drs. Cronyn and Lothrop made addresses introductory to the course in the absence of Dr. Buswell, who had been appointed to deliver the opening lecture. The laboratories of this college have been reconstructed and newly equipped with modern appliances of the most approved order and a prosperous year is indicated.

